# Water Shortage Strongly Alters Formation of Calcium Oxalate Druse Crystals and Leaf Traits in *Fagopyrum esculentum*

**DOI:** 10.3390/plants9070917

**Published:** 2020-07-20

**Authors:** Alenka Gaberščik, Mateja Grašič, Katarina Vogel-Mikuš, Mateja Germ, Aleksandra Golob

**Affiliations:** 1 Biotechnical Faculty, University of Ljubljana, Jamnikarjeva 101, SI-1000 Ljubljana, Slovenia; alenka.gaberscik@bf.uni-lj.si (A.G.); mateja.grasic@bf.uni-lj.si (M.G.); Katarina.VogelMikus@bf.uni-lj.si (K.V.-M.); aleksandra.golob@bf.uni-lj.si (A.G.); 2 Jožef Stefan Institute, Jamova 39, SI-1000 Ljubljana, Slovenia

**Keywords:** *Fagopyrum esculentum* Moench, water shortage, calcium oxalate druse crystals, leaf traits, element analysis

## Abstract

Common buckwheat (*Fagopyrum esculentum* Moench) is a robust plant with high resistance to different environmental constraints. It contains high levels of calcium oxalate (CaOx) druse crystals, although their role remains obscure. The objective was to examine the effects of water shortage on plant biomass partition and leaf traits and formation of CaOx druse crystals in common buckwheat. Buckwheat plants were exposed to favorable and reduced water availability for 28 days. The element composition and morphological, biochemical, physiological and optical traits of the leaves, and the plant biomass were investigated under these conditions. Measurements of photochemical efficiency of photosystem II showed undisturbed functioning for buckwheat exposed to water shortage, apparently due to partially closed stomata and more efficient water regulation. Strong relationships were seen between water-related parameters and Ca, Mn and S content, and size and density of CaOx druse crystals. Redundancy analysis revealed the importance of the size of CaOx druse crystals to explain reflection in the UV range. Water shortage resulted in shorter plants with the same leaf mass (i.e., increased mass:height ratio), which, together with denser leaf tissue and higher content of photosynthetic pigments and protective substances, provides an advantage under extreme weather conditions.

## 1. Introduction

Changes in the global climate are resulting in more pronounced droughts, which have a deleterious effect on global crop production and might compromise food security in the world [1]. Li et al. [2] reported that due to drought events, crop yields are likely to show reductions of >50% by 2050, and potentially by almost 90% by 2100. Yields of up to 50% can be lost if crops are subjected to drought during their reproductive phase [3].

Adaptive responses to drought stress are generally seen as alterations in plant phenotype and morphology due to changes in gene expression [4]. During their evolution, plants have acquired a variety of morphological and physiological traits that allow them to resist drought stress and physical damage to the plant [5], as in addition to drought, plants also need to resist extreme events such as heavy precipitation and strong winds [6]. The strategies that plants use to survive water shortage involve drought escape, drought avoidance and drought tolerance [5]. The first response of any plant drought avoidance strategy is closure of the stomata, which prevents excess loss of water [7]. In addition, plants respond to drought stress through various other morphological and physiological responses [8]. To cope with the present and future climate limitations and to assure food security, there is a need for production of more climate-resistant crops.

Buckwheats (*Fagopyrum* spp.) show high resistance to different environmental constraints, including drought [9,10,11,12,13]. Almost all parts of buckwheat plants represent sources for many compounds that can provide health benefits [14]. A primary example is the flavonoids, which are components of a variety of nutraceutical, pharmaceutical, medical and cosmetic formulations, due to their anti-oxidative, anti-inflammatory, anti-mutagenic and anti-carcinogenic properties [15]. *Fagopyrum* spp. plants also contain high levels of crystalline calcium oxalate (CaOx) deposits [16]. CaOx crystals in plants generally consist of either the monohydrate whewellite form or the dihydrate weddellite form [17], whereby formation of these crystals is usually associated with membranes, chambers or inclusions within the cell vacuole.

Calcium crystals have multiple functions in plants. As well as Ca regulation, these Ca crystals provide an internal reservoir for Ca, as they can be reabsorbed. They also serve as defense against herbivory, as they can substitute cell wall sclerification [18] and promote lignin polymerization [19], and they can also affect stomatal functioning. In buckwheat, it has also been shown that CaOx druse crystals can provide protection against solar radiation and against aluminum toxicity [16,20]. The potential roles of these CaOx druse crystals will also depend on the distributional patterns of these crystals [18]. In plants exposed to an insufficient supply of water, the availability of Ca might be limited, as the uptake of many elements in plants depends on transpiration stream [21,22,23].

Common buckwheat (*Fagopyrum esculentum* Moench) is an annual herbaceous plant that belongs to the family Polygonaceae [24]. In particular, it shows high resistance to various environmental stressors, including water shortage [25]. It also contains high levels of crystalline CaOx, which can have multiple roles in plants [18], including the role in shaping plant optical properties [16]. In the present study, we concentrated on the effects of moderate water shortage on common buckwheat stature and leaf traits, and included the presence of CaOx druse crystals and some of the main elements found in common buckwheat. We hypothesized that water shortage will affect CaOx druse crystal formation, to result in different numbers and sizes of these druse crystals, while also causing alterations to the physical and biochemical structure of the leaves, and their element composition, optical properties and vitality.

## 2. Material and Methods

### 2.1. Experimental Design

This study was conducted from May to July 2018 at the Biotechnical Faculty, University of Ljubljana, Slovenia (46°35′ N, 14°55′ E; 298 m a.s.l.). On 23 May 2018, seeds of common buckwheat (*Fagopyrum esculentum* Moench; cv. Darja) were sown in 14 plastic pots (dimensions, 45 × 45 × 36 cm) that were filled with soil from the Ljubljana Botanical Garden. This soil was classified as loamy (i.e., 49% sand, 37% silt, 14% clay). The further soil characteristics were: pH, 7.1; available P, 0.76 mg g^−1^; available K, 0.4 mg g^−1^; electrical conductivity, 0.23 mS cm^−1^; carbonates, 14.5%; C, 10.9%; N, 0.74%; C/N ratio, 14.9; organic matter, 18.1%; total C, 12.63%; cation exchange capacity, 0.41 mmol_c_ g^−1^; Ca^2+^, 0.34 mmol_c_ g^−1^; Mg^2+^, 0.062 mmol_c_ g^−1^; K^+^, 0.94 mmol_c_ (100 g)^−1^; Na^+^, 0.08 mmol_c_ (100 g)^−1^; total exchangeable bases, 0.42 mmol_c_ g^−1^; and base saturation, 99%.

**Figure 1:**
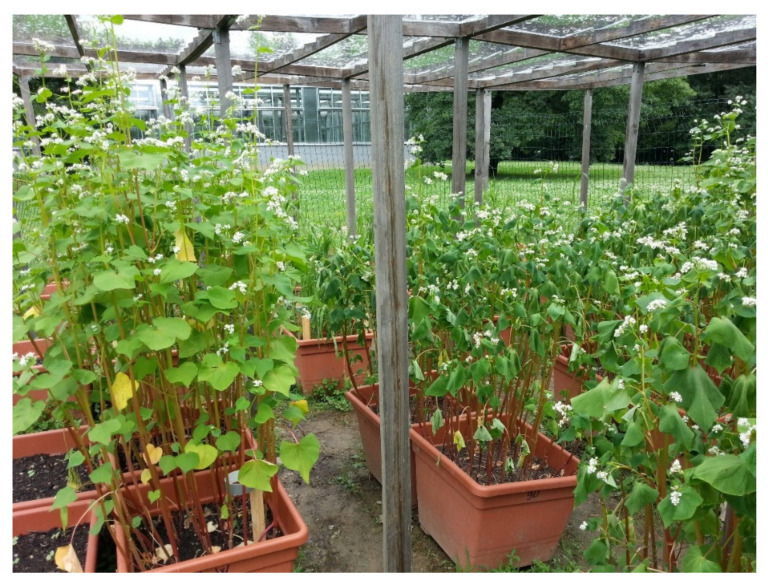
Experimental plants at 24 days from the start of the experiment. Left, control; right, water shortage.

On 4 June 2018, redundant plants were removed from the pots to provide 36 plants per pot. From 11 June 2018 onwards, 7 pots received regular watering (control), while 7 pots received one-third of the regular watering (water shortage). These pots were placed randomly under polycarbonate panels positioned outdoors, which were transparent to UV and visible radiation (transmission wavelength, ≥290 nm), to protect the experiment set-up from rain. In 8 days, soil moisture in the water shortage pots dropped to approximately 33% of that in the control pots. This difference in soil moisture was maintained for 28 days (Figure 1).

The soil moisture levels were measured with a moisture probe meter (MPM-160-B; ICT International Pty Ltd., Armidale, NSW, Australia). The soil moisture at the beginning of the experiment was determined at the beginning of the experimental period (21%), on 11 June 2018 (day 1). Afterwards, the soil moisture was measured on days 3, 6, 8, 9, 10, 14, 17, 20 and 28 of the experimental period.

The soil temperatures were recorded every 2 h from 11 June to 9 July 2018, using temperature data loggers (UTBI-001 TidbiT v2; Onset Computer Corporation, Bourne, MA, USA). The sensors were placed approximately 5 cm deep in the soil. Transfer of the data to the computer was done using a USB connector, using a base station (U-4 HOBO Optic USB; Onset Computer Corporation, Bourne, MA, USA) and a two-dimensional coupler (Onset Computer Corporation, Bourne, MA, USA). The meteorological conditions for the full experimental period (i.e., mean daily temperatures, wind, relative humidity, rainfall, solar irradiation) are presented in Table S1.

Sampling of the plants for the biochemical, physiological, optical, morphological and element analysis was performed for seven pots per each treatment. Three plants from each pot (subsamples) were taken at random.

### 2.2. Biochemical Analysis

The chlorophyll and carotenoid contents were determined according to Lichtenthaler and Buschmann [26,27]. The methodology of measuring anthocyanin contents followed the procedure reported by Drumm and Mohr [28]. Fresh leaves were homogenized in a mortar for anthocyanin extraction (extraction medium, 37% HCl: methanol; 1:99 [*v/v*]). The absorbance of the extracts was measured at 530 nm using a UV/VIS spectrometer (Lambda 12; Perkin–Elmer, Norwalk, CT, USA), with the anthocyanin contents calculated as absorbance per leaf area.

The methanol-soluble UV-B and UV-A–absorbing compounds were determined as described by Caldwell [29]. The UV-absorbing compounds were extracted from the homogenized fresh leaves using methanol: distilled water: 37% HCl (79:20:1 [*v/v/v*]). After that, the samples were centrifuged (2-16 PK; Sigma, Darmstadt, Germany), and extinction of the supernatants was measured from 280 nm to 400 nm at intervals of 1 nm, using a UV/VIS spectrometer (Lambda 12; Perkin–Elmer, Norwalk, CT, USA). The absorbances were determined from 280 nm to 320 nm for the UV-B–absorbing compounds and from 320 nm to 400 nm for the UV-A–absorbing compounds, and are calculated as absorbance per leaf area.

### 2.3. Physiological Analysis

The photochemical efficiency of the control and treated plants was determined from the fluorescence of chlorophyll in a photosystem (PS) II, as measured with a portable chlorophyll fluorometer (PAM-2100; Heinz Walz GmbH, Effeltrich, Germany). The potential photochemical efficiency of PS II was calculated according to Equation (1):

F_v_/F_m_ = (F_m_ − F_0_)/F_m_ (1)

where F_0_ and F_m_ are the minimal and maximal chlorophyll a fluorescence yields in the leaves, using clips for dark adaptation, and F_v_ is the variable fluorescence [30].

The respiratory potential of the mitochondria was estimated via the activity of the electron transport system (ETS) following the method proposed by Packard [31], as modified by Kenner and Ahmed [32]. Fresh leaf samples were collected and homogenized in a mortar in cold buffer (0.1 M sodium phosphate, 75 µM MgSO_4_, polyvinyl pyrrolidone, Triton X-100) and with an ultrasound homogenizer (Sono Pulse, GM-mini20; Bandelin electronic, Berlin, Germany); they were then centrifuged at 8500× *g* for 4 min at 0 °C in a top-refrigerated ultracentrifuge (2-16 PK; Sigma, Germany). Substrate buffer (0.1 M sodium phosphate, pH 8.4, 1.7 mM NADH, 0.25 mM NADPH, Triton X-100) containing 2 mg mL^−1^ 2-p-iodo-phenyl 3-p-nitrophenyl 5-phenyl tetrazolium chloride (INT) was added to the supernatant. The mixture was incubated for 40 min at 20 °C. The INT was used as the electron acceptor, whereby it was reduced to formazan, the absorbance of which was measured at 490 nm. The ETS activity was calculated from the rate of INT reduction, which was converted into the amount of oxygen used per g leaves, as dry mass (DM) per hour (μL O_2_ mg^−1^ leaf DW h^−1^).

Stomatal conductance was measured using a steady-state leaf porometer (Decagon Devices, Inc., Pullman, WA, USA), which measured the rate of water vapor diffusion via the leaf surfaces (mmol H_2_O m^−2^ s^−1^).

### 2.4. Optical Measurements

The light reflectance and transmittance spectra of fresh buckwheat leaves were measured in the laboratory immediately after their collection in the field. The procedure was as described in Klančnik et al. [33]. The measurements were carried out from 300 nm to 820 nm, at every ~1.3 nm, using a portable spectrophotometer (Jaz Modular Optical Sensing Suite; Ocean Optics, Inc., Dunedin, FL, USA) connected to an optical fiber (QP600-1-SR-BX; Ocean Optics, Inc., Dunedin, FL, USA) and an integrating sphere (ISP-30-6-R; Ocean Optics, Inc., Dunedin, FL, USA). The samples were irradiated using a UV-VIS-near-infrared light source (DH-2000; Ocean Optics, Inc., Dunedin, FL, USA).

### 2.5. Morphological Measurements

The samples for observation under light microscopy were prepared using free-hand sectioning technique as described by Bondada [34]. We used the fresh, first fully developed leaves of plants in the phase of flowering. Leaf tissue was taken from the central part of the leaf, left and right from the main vein. The cross-sections were made using a new double-sided razor blade and transferred into a drop of a short-term preservation liquid (70% alcohol:glycerin; 3:2 [*v/v*]) until examination under light microscopy (CX41; Olympus, Tokyo, Japan) with a digital camera (XC30; Olympus, Tokyo, Japan) and using the CellSens software (Olympus, Tokyo, Japan). Five slices of each subsample were measured. The numbers and diameters of CaOx druse crystals were determined, along with the thicknesses of the leaves, their upper and lower epidermis, and their palisade and spongy mesophyll. The density of CaOx druse crystals was determined as the number of CaOx druse crystals per surface area of the leaf cross-section. The density and length of the leaf stomata on the upper and lower leaf surfaces were also determined. The density of stomata was determined as number of stomata per area of upper and lower epidermis. Epidermis thickness, stomata and diameter of CaOx druse crystals were obtained at 400× magnification, with 100× magnification used for the rest of the measurements. The specific leaf areas were calculated as leaf areas per dry mass, and tissue density as dry mass per leaf volume.

For evaluation of the dry mass of the plant parts, three buckwheat plants were taken at random from each pot and separated into the leaf, stem and flower fractions. These were all dried in a laboratory oven at 105 °C to constant weight. These data are expressed as g plant part per plant.

### 2.6. Leaf Bulk Element Analysis

The element contents in the buckwheat leaves collected on day 28 were determined for phosphorus (P), sulphur (S), chlorine (Cl), potassium (K), calcium (Ca), manganese (Mn), iron (Fe) copper (Cu) and zinc (Zn), using X-ray fluorescence spectrometry. The dried and powdered leaves (100 mg) were pressed into tablets using a pellet die and a hydraulic press. The primary excitation source for the analysis was ^55^Fe (25 mCi; Isotope Products Laboratories, Valencia, PA, USA). The emitted fluorescence radiation was collected using a Si drift diode detector (Amptek, Inc., Bedford, MA, USA) with a 12-μm-thick beryllium window. The energy resolution of the spectrometer at count rates <1000 cps was 140 eV at 5.9 keV. The X-ray fluorescence spectrometry analysis was conducted under vacuum and the samples were irradiated for 2000 s to obtain spectra with sufficient statistics [35]. Analysis of the X-ray spectra was performed using an iterative least-squares program, as included in the quantitative X-ray analysis system software package [36]. The element quantification from the measured spectra was performed using quantitative analysis of environmental samples based on the fundamental parameters [37]. The quality assurance for the element analysis was determined using standard reference materials: NIST SRM 1573a.

### 2.7. Statistical Analyses

Normal distributions of the data were tested using Shapiro–Wilk tests. Differences between the conditions were tested using t-tests. Spearman’s correlation analysis was performed to test the relationships between some of the measured parameters. The level for significance was accepted at *p* < 0.05. The SPSS Statistics software, version 20.0 (IBM, Armonk, NY, USA), was used for the calculations.

Redundancy analysis was used to explain the variability of the reflectance spectra and the parameters related to CaOx druse crystals according to the leaf biochemical, anatomical, morphological and element properties, as well as the water-related parameters. Monte Carlo permutation tests with 999 permutations were used to test the significance of the effects. Forward selection of the leaf traits was used to avoid co-linearity of the variables. The level for significance was accepted at *p* < 0.05. These analyses were performed using the Canoco software for Windows 5.0 [38].

## 3. Results

### 3.1. Growth Conditions and Leaf Traits

**Figure 2:**
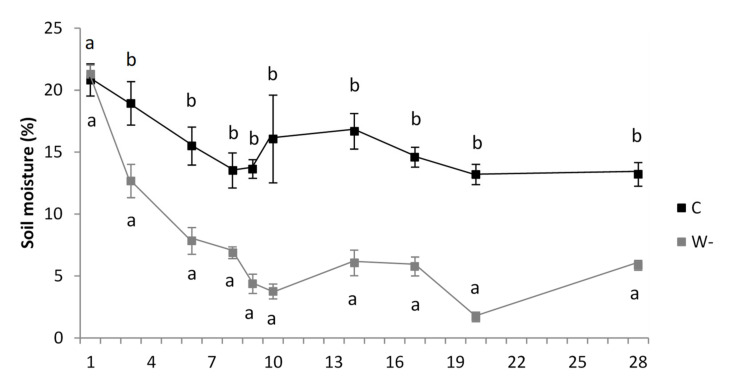
Soil moisture in the pots for the control (C) and water shortage (W-) treatments of the buckwheat plants. Data are means ± standard deviation (*n* = 7). Different letters indicate significant differences in soil moisture between treatments within each sampling day (*p* ≤ 0.05; *t*-tests).

Two significantly different soil moisture levels were created for the experimental period that was maintained for 28 days. Initially, the soil moisture levels did not differ between the control and water shortage treatments. Later, the differences in soil moisture gradually increased between the two treatments. Mean differences in soil moisture between treatments (for days 3, 6, 8, 9, 10, 14, 17, 20, 28) were 6.3%, 7.7%, 6.6%, 9.3%, 12.3%, 10.6%, 8.8%, 11.5% and 7.3%, respectively (Figure 2). There were no significant differences between the two water treatments for soil temperature at a depth of 5 cm. Mean soil temperature ranged from 21.7 °C to 22.5 °C (data not shown).

Water availability affected most of the parameters that were measured for these buckwheat leaves (Table 1). Inspection of the transverse leaf sections revealed that compared to control plants, those exposed to water shortage had thinner leaves with thinner palisade and spongy mesophyll, although there were no differences in the thickness of the epidermis. The water shortage plants had significantly lower relative water content (RWC) and specific leaf area (SLA), but higher tissue density. Water shortage increased the number of stomata on the upper and lower epidermis, although the dimensions of the stomata were smaller. Water shortage also had a significant positive impact on the density of CaOx druse crystals in these buckwheat leaves, although the CaOx druse crystals were smaller. Additionally, despite the increased number of CaOx druse crystals compared to the control plants, these occupied a significantly smaller surface area of a leaf cross-section in the water shortage plants (1.78% vs. 1.29%; *p* = 0.021) (Table 1).

The biochemical leaf traits also showed significant differences between the control and water shortage plants. Compared to the control, the leaves of the water shortage plants had higher contents of photosynthetic pigments (total chlorophyll, carotenoids) as well as UV-A– and UV-B–absorbing compounds. However, there were no significant differences in the anthocyanin contents between the control and water shortage plants (Table 1).

**Figure 3:**
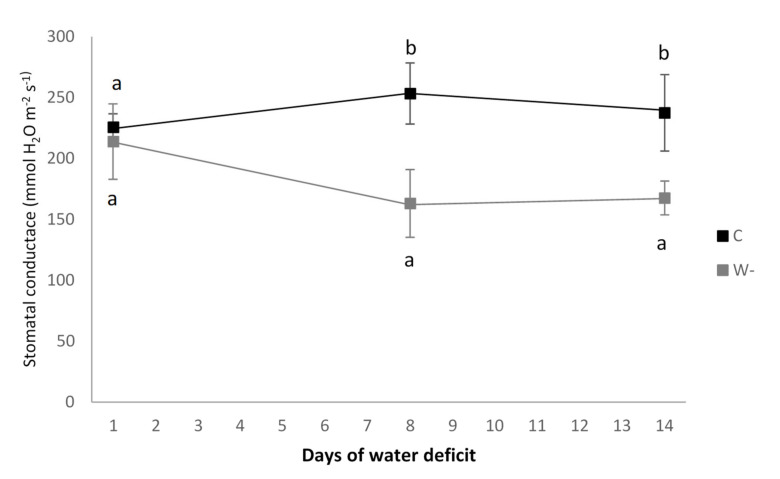
Stomatal conductance of the leaves for the control (C) and water shortage (W-) treatments of the buckwheat plants. Data are means ± standard deviation (*n* = 7). Different letters indicate significant differences in stomatal conductance between treatments within each sampling day (*p* ≤ 0.05; *t*-tests).

Although the plants were exposed to water shortage, their vitality was not affected. This is evident when comparing the control and water shortage leaves in terms of the maintained high values for both potential (F_v_/F_m_; 0.8 vs. 0.79) and effective photochemical efficiency of PS II (∆F_v_/F_m_’; 0.68 vs. 0.68). There were also no significant differences in the activity of the ETS of mitochondria between the control and water shortage plants (2.55 vs. 2.65 µL O_2_ mg^−1^ DM h^−1^). However, compared to the control plants, the plants exposed to water shortage showed significantly decreased stomatal conductance (Figure 3).

The element analysis of the buckwheat leaves indicated significant effects of water availability on Ca, Mn, K and S contents. The contents of the other elements measured in the buckwheat leaves did not differ between the control and water shortage plants (Table 2).

### 3.2. Plant Biomass

**Figure 4:**
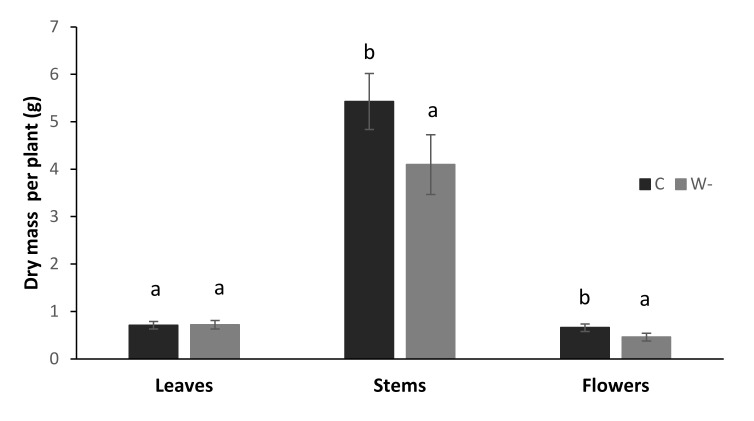
Dry mass per plant for the control (C) and water shortage (W-) treatments of the buckwheat plants. Data are means ± standard deviation (*n* = 7). Different letters indicate significant differences between treatments within each plant part (*p* ≤ 0.05; *t*-tests).

Water shortage significantly decreased the fresh (data not shown) and dry (Figure 4) masses of stems and flowers, although there were no significant differences for the leaves between the control and water shortage plants (Figure 4). These differences in biomass corresponded to the differences in plant heights across these conditions. The control plants were on average 30 cm higher than the water shortage plants (134 ± 5 vs. 104 ± 7 cm; *p* < 0.001). The ratios of leaf dry mass to plant height showed that compared to the control plants, those under water shortage had significantly higher leaf biomass:height ratio (5.87 ± 0.63 vs. 7.68 ± 0.75 g m^−1^; *p* < 0.001).

### 3.3. Relationships between Water Availability Indicators and Certain Leaf Traits

**Figure 5:**
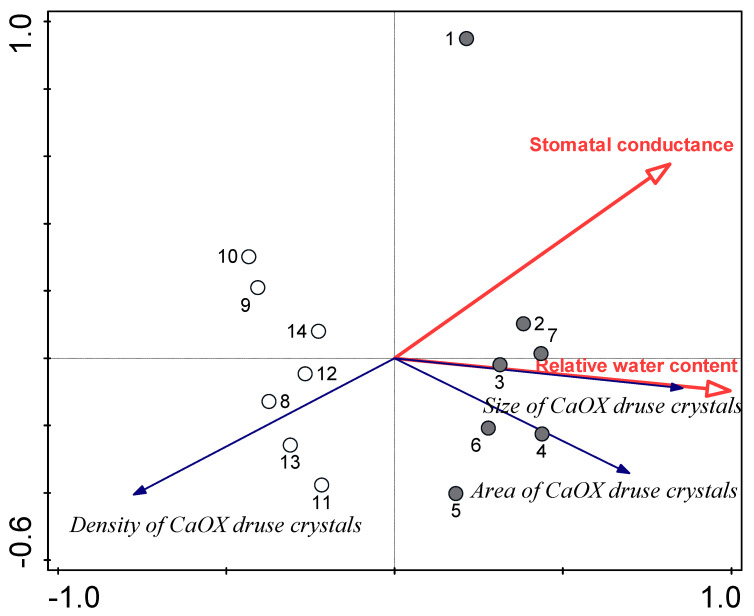
Redundancy analysis plot showing the strengths of the associations between the water status indicators, as relative water content and stomatal conductance, and CaOx druse crystal parameters for the buckwheat leaves. Filled circles, control; open circles, water shortage.

Redundancy analysis revealed strong positive relationships between the sizes and areas of CaOx druse crystals, and RWC and stomatal conductance, with negative relationships for these parameters for the density of CaOx druse crystals (Figure 5). For the variability of CaOx druse crystal parameters, RWC alone explained 60.1% and stomatal conductance alone 43.6%. When considered together here, the 60.1% explained RWC (*p* = 0.001) was increased by a further 10.1% by stomatal conductance (*p* = 0.047). The samples formed two distinct groups according to water availability (Figure 5).

**Figure 6:**
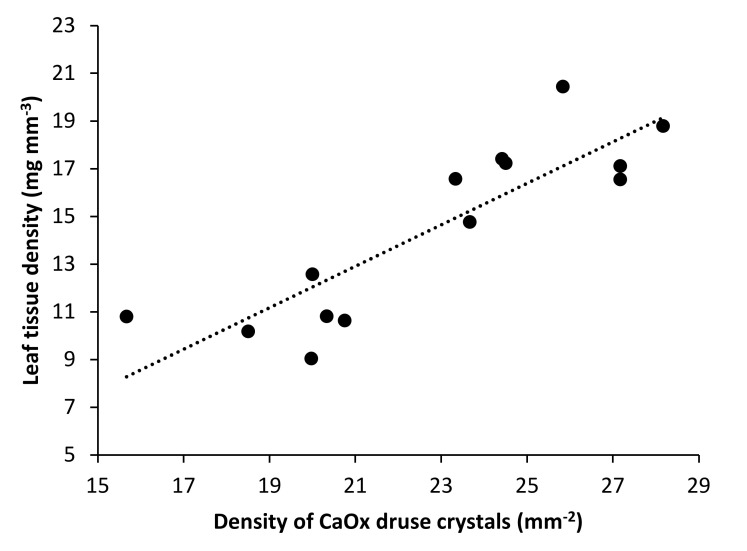
Regression graph showing strong positive relationship between leaf tissue density and density of CaOx druse crystals.

Additional redundancy analysis was carried out to explain the variability of CaOx druse crystal parameters according to the leaf morphological parameters. Here, there were positive associations of the thickness of palisade mesophyll with the size and area of CaOx druse crystals, and positive association of tissue density (as mass per leaf volume) with the density of CaOx druse crystals. The thickness of palisade mesophyll alone explained 69.7% of the variability of CaOx druse crystal parameters, and when considered together, the 62.7% explained by tissue density alone was increased to 69.7% by the thickness of palisade mesophyll (*p* = 0.001), with a further 10.4% of the variability explained by tissue density (*p* = 0.016) (see Figure S1). Tissue density was strongly negatively correlated to relative water content (−0.90, *p* ≤ 0.01) and positively to the density of CaOx druse crystals (0.83, *p* ≤ 0.01) (Figure 6).

**Figure 7:**
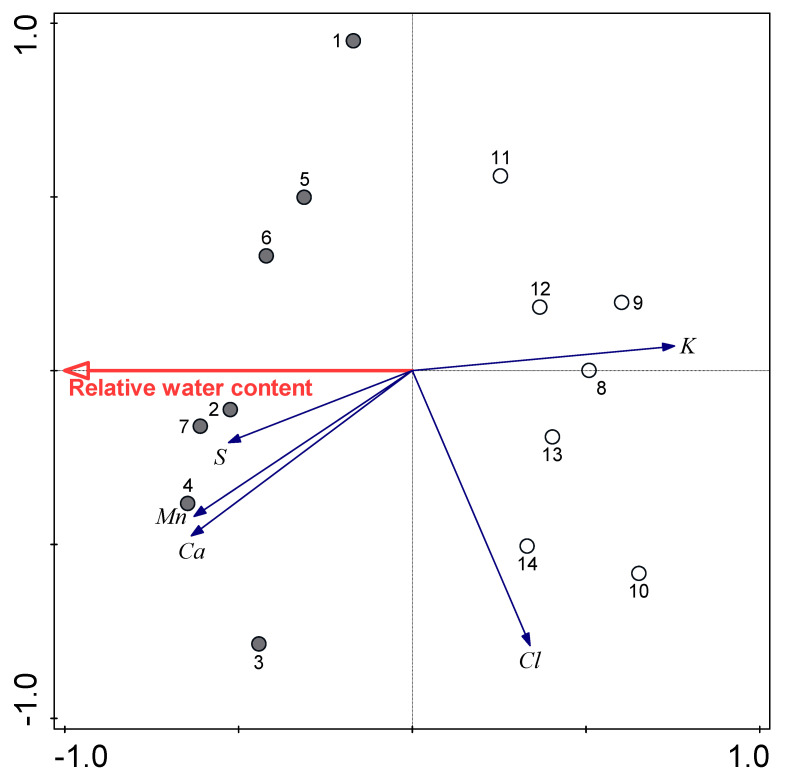
Redundancy analysis plot showing the strengths of associations between relative water content and element contents in the buckwheat leaves. Filled circles, control; open circles, water shortage.

Redundancy analysis was also used to examine the importance of RWC for the studied elements in the buckwheat leaves (Figure 7). There was positive association of RWC and leaf contents of Ca and Mn, and negative association for K and Cl. In total, RWC explained 35.5% of the variability of element contents in these buckwheat leaves (*p* = 0.001).

Correlation analysis showed strong relationships between water-related parameters and leaf structural properties. For example, relative water content was highly positively correlated to CaOx druse crystal diameter (0.88, *p* ≤ 0.01) and their total area (0.69, *p* ≤ 0.01), while strong negative correlation was obtained for density of CaOx druse crystals (−0.70, *p* ≤ 0.01).

### 3.4. Optical Properties

**Figure 8:**
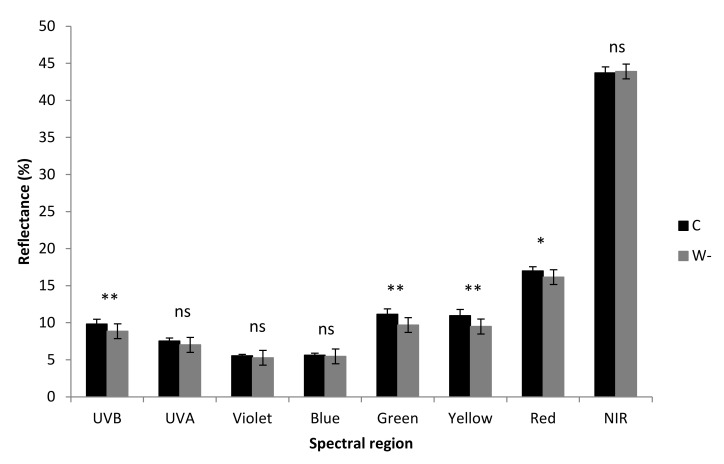
Leaf reflectance of the control (C) and water shortage (W-) treatments of the buckwheat plants. Data are means ± standard deviation (*n* = 7). *, *p* ≤ 0.05; **, *p* ≤ 0.01 (*t*-test); ns—not significant.

The reflectance spectra of the leaves from the control and water shortage buckwheat plants showed differences across various regions of the spectra (Figure 8, Figure S2A). Exposure to water shortage resulted in significant decreases in leaf reflectance for the UV-B, green, yellow and red regions of the spectrum. However, there were no significant differences in the transmittance spectra between the leaves of the control and water shortage plants (Figure S2B).

**Figure 9:**
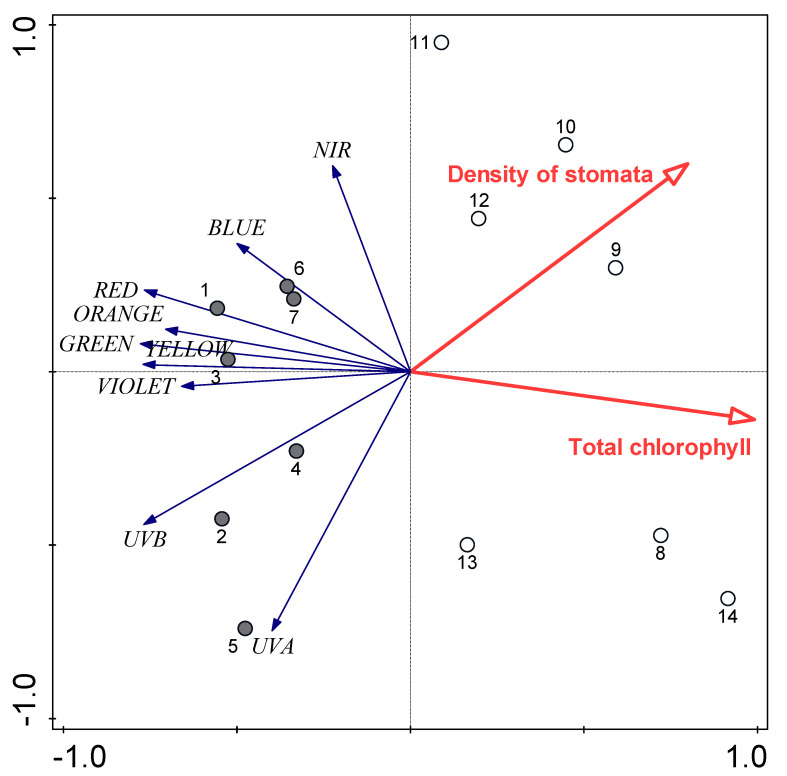
Redundancy analysis plot showing the strengths of the associations for total chlorophyll content and density of stomata with leaf reflectance spectra. Filled circles, control; open circles, water shortage.

Redundancy analysis was also used to explain the variability of the leaf reflectance spectra, which revealed that it had negative relationships with total chlorophyll content and density of stomata. Total chlorophyll content alone explained 41.4% of the variability of leaf reflectance spectra, and stomata density alone explained 32.1%. When considered together, the 41.4% explained by total chlorophyll content (*p* = 0.002) was further increased by 15.2% by stomata density (*p* = 0.024). Once again, the samples formed two separate groups according to these parameters (Figure 9).

If we considered leaf reflectance spectra according to the individual spectral regions, redundancy analysis revealed that the size of CaOx druse crystals explained 46.2% (*p* = 0.004) of the variability of the reflectance in the UV region. For the variability of the reflectance in the violet/blue regions and green/yellow regions, total chlorophyll content explained 35.9% (*p* = 0.017) and 58.6% (*p* = 0.002) of these, respectively. For the red/near-infrared regions of the spectrum, total chlorophyll content explained 35.9% (*p* = 0.002) of the variance, with an additional 24.6% for Ca content (*p* = 0.005) (Table 3).

## 4. Discussion

The buckwheat plants investigated here showed some strong responses to this moderate water shortage. These responses were partly beneficial, as they improved plant resistance to this constraint. Comparison of the potential and actual efficiencies of PS II of the plants showed that they did not suffer appreciable stress here due to water shortage, as the control and water shortage values were similar. Furthermore, the potential photochemical efficiencies under both of these treatments were close to optimal (0.83), again indicating the absence of any permanent stress [39,40,41]. However, photochemical efficiency demonstrated mild stress transitions in both the control and water shortage plants; this was possibly the consequence of midday depression [5] and reversible inactivation, rather than damage to the reaction centers. The F_v_/F_m_ values also indicated no drought-induced damage to the flow of electrons for PS II, as previously reported for potato [42,43], kidney bean [44], sunflower [45] and some other species [46]. The good physiological state of the plants was also supported by analysis of the respiratory potential, as measured by the ETS activity, which defines the general metabolic activity of organisms. Here, the ETS activity remained at the same level under both plant treatments, and thus the flow of electrons in the respiratory chain was not impaired [43].

This undisturbed functioning under the water shortage conditions appears to be due to only partial closing of the stomata, which would allow photosynthetic processes to continue. Stomata closure is usually one of the primary plant responses to dehydration to prevent additional water loss, and it is essential for the success of any drought-avoidance strategy of such plants [7]. RWC of the leaf tissue was significantly lower in the water shortage plants (67%) compared to the control plants (79%). In *Olea europea*, the stomata are completely closed at RWC of 78% [47], which might be related to the scleromorphic structure of the leaves in this species. In different plants that are not limited by the stomata, the photosynthetic rate remains relatively high at RWC of 50% to 70% [48].

An important feature observed in the water shortage plants was their increased leaf tissue density, which usually occurs along the dryness gradient [49]. This might be the consequence of the restriction of both cell division and cell expansion [50,51]. A study with *Arabidopsis* showed that the interactions among cell division, cell growth and intercellular spaces had a major influence on leaf photosynthetic performance [52]. Undisturbed plant functioning might also be related to the denser and smaller stomata in the water shortage plants, which would provide more efficient water regulation. It has been shown that the stomata guard cells are the end product of a specialized lineage that is dynamic and flexible, and that can alter the production of stomata in response to changes in the environment [53].

The absence of stress and denser mesophyll tissue resulted in increases in photosynthetic pigments and some of the other pigments, which included higher contents of UV-absorbing compounds. These UV-absorbing compounds are mainly represented by flavonoids, which are structurally diverse secondary metabolites with multiple functions [54]. They can act as unique UV-selective filters, helping to improve water management, regulate plant development, protect plants from different biotic and abiotic stresses, and they can also function as signaling molecules, allelopathic compounds, phytoalexins and detoxifying agents, and protect plants against pathogens and herbivores [55,56]. Flavonoid increases also occur in response to drought [57]. However, the signaling and regulation mechanisms of flavonoids in any stress mitigation mechanisms remain unclear [58].

A further consequence of water shortage was the difference in leaf element contents. These differences might lead to imbalances of nutrients, which would have strong effects on various growth and developmental processes [59]; this was also evident in the present study. Our previous study with cereals showed that leaf contents of Si, P, Ca and S decreased following water shortage, while leaf contents of Cl and K increased [21]. In the present study, element analysis of these buckwheat leaves indicated significant negative effects of water shortage on leaf contents of Ca, Mn, K and S, but not on P. The latter element was not affected; this might be a consequence of species-specific responses. Element uptake depends on different parameters, such as root interception, mass flow through leaf transpiration, diffusion and chelation [60]. Grašič et al. [22,23] reported that the contents of elements appeared to be related to leaf stomatal conductance. Indeed, in the present study, RDA showed positive relationships between Ca, Mn and S contents, and RWC, while this relation for Cl and K contents was negative. Hussain et al. [59] also showed that drought stress can reduce the uptake of minerals in sunflower. They claimed that reduced mineralization rates due to low moisture availability and limited translocation of minerals to shoots were the primary mechanisms for reduced mineral uptake.

The decrease in Ca uptake in the water shortage buckwheat plants resulted in changes in the number and size of CaOx druse crystals. These are known to have multiple functions, and they were more numerous and smaller in the water shortage plants. This was expected, as it has been shown that oxalate synthesis in plant tissues increases with increased calcium supply and uptake [18]. RDA explained a large portion of the variability of the druse crystal parameters in terms of plant stomatal conductance and RWC. The various parameters of CaOx druse crystals were also related to leaf morphology, as the density of these druse crystals was positively related to tissue density, while the relation was negative for their size and total area per leaf transection. However, the variability of the properties of CaOx druse crystals was limited, as genetic regulation of CaOx formation promotes constancy of the crystal morphology within species, cell specialization, crystal growth and cell expansion [61].

The efficiency of solar energy harvesting in the leaves depends on their optical properties, which are related to their biophysical structure [62]. Leaf optical properties can be estimated by measurements of the light they reflect, absorb and/or transmit [63]. In the present study, leaf reflectance, but not leaf transmittance, was significantly affected by water shortage, with consequent changes in the biophysical structure, including for the leaf biochemical traits and the CaOx druse crystal properties. The RDA analysis to define reflectance in the different spectral regions with different parameters revealed the importance of CaOx druse crystals in explaining reflection in the UV range, and the content of Ca in explaining the red and near-infrared reflection, with chlorophyll content explaining most of the variability for all of the regions, except for UV. A previous study has already shown that CaOx druse crystals might have an important role in reflecting light in the UV region while transmitting visible regions [16].

Plant resilience to extreme environmental events is crucial for their survival in the future [64]. Gutschick and BassiriRad [65] reported that the most important traits for plant resilience are those that increase resource acquisition and use efficiency. In the present study, the overall influence of water shortage was for shorter plants with the same leaf biomass, which therefore increased their leaf mass:height ratio. Along with denser leaf tissue, this presents an advantage under extreme conditions, such as heavy rain, winds and prolonged droughts, as it can prevent tissue damage and plant lodging, while high levels of photosynthetic and protective pigments can promote their functioning.

The production of CaOx druse crystals is a dynamic, reversible process. When Ca concentrations in the plant are reduced, as was the case in our study, these crystals may dissolve [66], which assures normal functioning of the plant, including stress alleviation during drought [67].

## Figures and Tables

**Table 1:** Morphological, biochemical and physiological traits of the buckwheat leaves grown under the control and water shortage conditions.

Leaf Trait	Treatment		*p*
	Control	Water Shortage	
**Anatomical and morphological**			
Relative water content (%)	79.5 ± 2.33 ^b^	67.1 ± 2.04 ^a^	<0.001
Specific leaf area (cm^2^ mg^−1^ DM)	0.34 ± 0.04 ^b^	0.30 ± 0.2 ^a^	0.049
Tissue density (mg mm^−3^)	11.3 ± 1.9 ^a^	17.7 ± 1.4 ^b^	<0.001
Leaf thickness (µm)	275.4 ± 31.0 ^b^	191.8 ± 6.3 ^a^	<0.001
Palisade mesophyll (µm)	136.1 ± 22.0 ^b^	88.4 ± 5.5 ^a^	<0.001
Spongy mesophyll (µm)	85.9 ± 10.7 ^b^	59.5 ± 3.5 ^a^	<0.001
Density of CaOx druse crystals (mm^−2^)	19.8 ± 2.4 ^a^	25.8 ± 1.9 ^b^	<0.001
Diameter of CaOx druse crystals (µm)	33.3 ± 4.3 ^b^	25.2 ± 1.4 ^a^	<0.001
Area of CaOx druse crystals (% transection area)	1.78 ± 0.46 ^b^	1.29 ± 0.17 ^a^	0.021
***Adaxial leaf surface***			
Epidermis thickness (µm)	29.6 ± 3.0 ^a^	27.5 ± 5.2 ^a^	0.115
Stomata density (mm^−2^)	20.9 ± 3.4 ^a^	31.7 ± 4.0 ^b^	<0.001
Stomata length (µm)	35.8 ± 2.0 ^b^	30.4 ± 1.0 ^a^	<0.001
Stomata width (µm)	23.0 ± 0.8 ^b^	21.7 ± 1.2 ^a^	0.033
***Abaxial surface***			
Epidermis thickness (µm)	22.9 ± 2.2 ^a^	21.7 ± 1.2 ^a^	0.209
Stomata density (mm^−2^)	50.0 ± 13.6 ^a^	94.7 ± 9.7 ^b^	<0.001
Stomata length (µm)	34.2 ± 2.2 ^b^	28.3 ± 1.2 ^a^	<0.001
Stomata width (µm)	21.9 ± 0.7 ^b^	20.1 ± 1.1 ^a^	0.003
**Biochemical**			
Total chlorophyll (mg dm^−2^)	3.16 ± 0.26 ^a^	5.37 ± 0.95 ^b^	<0.001
Carotenoids (mg dm^−2^)	0.49 ± 0.10 ^a^	0.74 ± 0.09 ^b^	<0.001
Anthocyanins (rel. units cm^−2^)	0.76 ± 0.06 ^a^	0.70 ± 0.13 ^a^	0.357
UV-B–absorbing compounds (rel. units cm^−2^)	160 ± 23 ^a^	232 ± 27 ^b^	<0.001
UV-A–absorbing compounds (rel. units cm^−2^)	314 ± 60 ^a^	495 ± 60 ^b^	<0.001
**Physiological**			
Stomatal conductance (mmol H_2_O m^−2^ s^−1^)	253 ± 25 ^b^	163 ± 28 ^a^	<0.001
F_v_/F_m_	0.8 ± 0.02 ^a^	0.79 ± 0.01 ^a^	0.780
∆F_v_/F_m_’	0.68 ± 0.02 ^a^	0.67 ± 0.02 ^a^	0.414
ETS (µL O_2_ mg^−1^ DM h^−1^)	2.55 ± 0.44 ^a^	2.65 ± 0.25 ^a^	0.605

Data are means ± standard deviation. Means followed by different superscript letters are significantly different at *p* < 0.05 (*n* = 7, *t*-test). DM, dry matter; CaOx, calcium oxalate; ETS, electron transport system.

**Table 2:** Contents of the elements in the buckwheat leaves grown under the control and water shortage conditions.

Element	Element Contents (mg g^−1^ DM) according to Treatment		*p*
	Control	Water Shortage	
Phosphorus	2.34 ± 0.38 ^a^	2.50 ± 0.49 ^a^	0.513
Sulphur	0.51 ± 0.15 ^b^	0.38 ± 0.07 ^a^	0.049
Chlorine	9.42 ± 4.15 ^a^	12.94 ± 2.71 ^a^	0.084
Potassium	12.86 ± 2.13 ^a^	21.80 ± 4.79 ^b^	0.001
Calcium	38.98 ± 3.56 ^b^	32.22 ± 4.71 ^a^	0.011
Manganese	0.13 ± 0.03 ^b^	0.10 ± 0.02 ^a^	0.048
Iron	0.27 ± 0.02 ^a^	0.28 ± 0.03 ^a^	0.707
Copper	0.016 ± 0.004 ^a^	0.017 ± 0.004 ^a^	0.620
Zinc	0.067 ± 0.01 ^a^	0.078 ± 0.016 ^a^	0.181

Data are means ± standard deviation. Means followed by different superscript letters are significantly different at *p* < 0.05 (*n* = 7; *t*-test). DM, dry matter.

**Table 3:** Explained variance of the reflectance in different spectral regions for the buckwheat leaf surface defined by leaf traits, as obtained by redundancy analysis.

Spectral Region	Leaf Traits	Explained Variance (%)	*p*
UV	Size of CaOx druse crystals	46.2	0.004
Violet, blue	Total chlorophyll	35.9	0.017
	Calcium content	18.4	0.034
Green, yellow	Total chlorophyll	58.6	0.002
Red, near-infrared	Total chlorophyll	35.9	0.002
	Calcium content	24.6	0.005

CaOx, calcium oxalate.

## Supplementary Materials

The following are available online at https://www.mdpi.com/2223-7747/9/7/917/s1, Figure S1: RDA plot showing the strength of associations of thickness of palisade mesophyll and tissue density with druses of CaOX crystals parameters. Dark grey circles, control; white circles, water shortage, Figure S2: Reflectance (A) and transmittance (B) spectra of control and water-deprived buckwheat leaves. C, control; W-, water shortage, Table S1: Meteorological conditions during the field experiment.

## References

[1] Raza A., Razzaq A., Mehmood S.S., Zou X., Zhang X., Lv Y., Xu J. (2019). Impact of climate change on crop adaptation and strategies to tackle its outcome: A review. Plants.

[2] Li Y., Ye W., Wang M., Yan X. (2009). Climate change and drought: A risk assessment of crop-yield impacts. Clim. Res..

[3] Venuprasad R., Lafitte H., Atlin G. (2007). Response to direct selection for grain yield under drought stress in rice. Crop. Sci..

[4] Aghaie P., Tafreshi S.A.H., Ebrahimi M.A., Haerinasaba M. (2018). Tolerance evaluation and clustering of fourteen tomato cultivars grown under mild and severe drought conditions. Sci. Hortic..

[5] Larcher W. (2003). Physiological Plant Ecology: Ecophysiology and Stress Physiology of Functional Groups.

[6] Kasajima S., Hatae K., Morishita T. (2017). Seed-setting habit of a semi-dwarf common buckwheat line. Fagopyrum.

[7] Osmolovskaya N., Shumilina J., Kim A., Didio A., Grishina T., Bilova T., Keltsieva O.A., Zhukov V., Tikhonovich I., Tarakhovskaya E. (2018). Methodology of drought stress research: Experimental set-up and physiological characterization. Int. J. Mol. Sci..

[8] Chen D., Wang S., Cao B., Cao D., Leng G., Li H., Yin L., Shan L., Deng X. (2016). Genotypic variation in growth and physiological response to drought stress and re-watering reveals the critical role of recovery in drought adaptation in maize seedlings. Front. Plant Sci..

[9] Gaberščik A., Vončina M., Trošt Sedej T., Germ M., Björn L.O. (2002). Growth and production of buckwheat (*Fagopyrum esculentum*) treated with reduced, ambient, and enhanced UV-B radiation. J. Photochem. Photobiol. B Biol..

[10] Breznik B., Germ M., Gaberščik A., Kreft I. (2005). Combined effects of elevated UV-B radiation and the addition of selenium on common (*Fagopyrum esculentum* Moench) and tartary (*Fagopyrum tataricum* (L.) Gaertn.) buckwheat. Photosyntetica.

[11] Dolinar N., Germ M., Kreft I., Breznik B., Gaberščik A. (2007). Effects of water deficit and selenium on common buckwheat (*Fagopyrum esculentum* Moench.) plants. Photosyntetica.

[12] Germ M., Breznik B., Dolinar N., Kreft I., Gaberščik A. (2013). The combined effect of water limitation and UV-B radiation on common and tartary buckwheat. Cereal Res. Commun..

[13] Golob A., Kugovnik A., Kreft I., Gaberščik A., Germ M. (2019). The interactions between UV radiation, drought and selenium in different buckwheat species. Acta Biol. Slov..

[14] Musilová J., Lachman J., Bystrická J., Vollmannová A., Čičová I., Timoracká M. (2013). Cultivar and growth phases–the factors affecting antioxidant activity of buckwheat (*Fagopyrum esculentum* Moench.). Acta Agric. Slov..

[15] Panche A.N., Diwan A.D., Chandra S.R. (2016). Flavonoids: An overview. J. Nutr. Sci..

[16] Golob A., Stibilj V., Nečemer M., Kump P., Kreft I., Hočevar A., Gaberščik A., Germ M. (2018). Calcium oxalate druses affect leaf optical properties in selenium-treated Fagopyrum tataricum. J. Photochem. Photobiol. B Biol..

[17] Franceschi V.R., Horner H.T. (1980). Calcium oxalate crystals in plants. Bot. Rev..

[18] Webb M.A. (1999). Cell-mediated crystallization of calcium oxalate in plants. Plant Cell..

[19] Ruiz L.P., Mansfield T.A. (1994). A postulated role for calcium oxalate in the regulation of calcium ions in the vicinity of stomatal guard cells. New Phytol..

[20] Kreft I., Zhou M., Golob A., Germ M., Likar M., Dziedzic K., Luthar Z. (2019). Breeding buckwheat for nutritional quality. Breed. Sci..

[21] Grašič M., Malovrh U., Golob A., Vogel-Mikuš K., Gaberščik A. (2019). Effects of water availability and UV radiation on silicon accumulation in the C4 crop proso millet. Photochem. Photobiol. Sci..

[22] Grašič M., Dobravc M., Golob A., Vogel-Mikuš K., Gaberščik A. (2019). Water shortage reduces silicon uptake in barley leaves. Agric. Water Manag..

[23] Grašič M., Golob A., Vogel-Mikuš K., Gaberščik A. (2019). Severe water deficiency during the mid-vegetative and reproductive phase has little effect on proso millet performance. Water.

[24] Martinčič A., Wraber T., Jogan N., Podobnik A., Turk B., Vreš B., Ravnik V., Frajman B., Strgulc Krajšek S., Trčak B. (2007). Mala flora Slovenije–Ključ za Določanje Praprotnic in Semenk.

[25] Halbrecq B., Romedenne P., Ledent J.F. (2005). Evolution of flowering, ripening and seed set in buckwheat (*Fagopyrum esculentum* Moench): Quantitative analysis. Eur. J. Agron..

[26] Lichtenthaler H.K., Buschmann C. (2001). Extraction of photosynthetic tissues: Chlorophylls and carotenoids. Curr. Protoc. Food Anal. Chem..

[27] Lichtenthaler H.K., Buschmann C. (2001). Chlorophylls and carotenoids: Measurement and characterization by UV-VIS spectroscopy. Curr. Protoc. Food Anal. Chem..

[28] Drumm H., Mohr H. (1978). The mode of interaction between blue (UV) light photoreceptor and phytochrome in anthocyanin formation of the *Sorghum* seedling. Photochem. Photobiol..

[29] Caldwell M.M. (1968). Solar ultraviolet radiation as an ecological factor for alpine plants. Ecol. Monogr..

[30] Schreiber U., Kühl M., Klimant I., Reising H. (1996). Measurement of chlorophyll fluorescence within leaves using a modified PAM fluorometer with a fiber-optic microprobe. Photosynth. Res..

[31] Packard T.T. (1971). The measurement of respiratory electron transport activity in marine phytoplankton. J. Mar. Res..

[32] Kenner A.A., Ahmed S.I. (1975). Measurements of electron transport activities in marine phytoplankton. Mar. Biol..

[33] Klančnik K., Vogel-Mikuš K., Gaberščik A. (2014). Silicified structures affect leaf optical properties in grasses and sedge. J. Photochem. Photobiol. B.

[34] Bondada B.R. (2012). An array of simple, fast, and safe approaches to visualizing fine cellular structures in free-hand sections of stem, leaf, and fruit using optical microscopy. Curr. Bot..

[35] Nečemer M., Kump P., Ščančar J., Jaćimović R., Simčič J., Pelicon P., Budnar M., Jeran Z., Pongrac P., Regvar M. (2008). Application of X-ray fluorescence analytical techniques in phytoremediation and plant biology studies. Spectrochim. Acta Part B At. Spectrosc..

[36] Vekemans B., Janssens K., Vincze L., Adams F., Vanespen P. (1994). Analysis of X-ray spectra by iterative least squares (AXIL): New developments. X-ray Spectrom..

[37] Kump P., Nečemer M., Rupnik Z., Pelicon P., Ponikvar D., Vogel-Mikuš K., Regvar M., Pongrac P. (2011). Improvement of the XRF quantification and enhancement of the combined applications by EDXRF and micro-PIXE. Integration of Nuclear Spectrometry Methods as a New Approach to Material Research.

[38] ter Braak C.J.F., Šmilauer P. (2002). CANOCO Reference Manual and CanoDraw for Windows Userʹs Guide: Software for Canonical Community Ordination (Version 4.5).

[39] Björkman O., Demmig B. (1987). Photon yield of O_2_ evolution and chlorophyll fluorescence characteristics at 77 K among vascular plants of diverse origins. Planta.

[40] Pfündel E. (1998). Estimating the contribution of photosystem I to total leaf chlorophyll fluorescence. Photosyn. Res..

[41] Urban L., Aarrouf J., Bidel L.P.R. (2017). Assessing the effects of water deficit on photosynthesis using parameters derived from measurements of leaf gas exchange and of chlorophyll a fluorescence. Front. Plant Sci..

[42] Tourneux C., Peltier G. (1995). Effect of water deficit on photosynthetic oxygen exchange measured using 18O_2_ and mass spectrometry in *Solanum tuberosum* L. leaf discs. Planta.

[43] Germ M., Kreft I., Stibilj V., Urbanc-Berčič O. (2007). Combined effects of selenium and drought on photosynthesis and mitochondrial respiration in potato. Plant Physiol. Biochem..

[44] Miyashita K., Tanakamaru S., Maitani T., Kimura K. (2005). Recovery responses of photosynthesis, transpiration, and stomatal conductance in kidney bean following drought stress. Environ. Exp. Bot..

[45] Pankovic D., Sakac Z., Kevres S., Plesnicar M. (1999). Acclimation to long-term water deficit in the leaves of two sunflower hybrids: Photosynthesis, electron transport and carbon metabolism. J. Exp. Bot..

[46] Chaves M.M., Pereira J.S., Maroco J.P., Rodrigues M.L., Picardo C.P.P., Osorio M.L., Carvalho I., Faria T., Pinheiro C. (2002). How plants cope with water stress in the field. Photosynthesis and growth. Ann. Bot..

[47] Sun P., Wahbi S., Tsonev T., Haworth M., Liu S., Centritto M. (2014). On the use of leaf spectral indices to assess water status and photosynthetic limitations in Olea europaea L. during water stress and recovery. PLoS ONE.

[48] Kaiser W.M. (1987). Effects of water deficit on photosynthetic capacity. Physiol. Plant..

[49] de la Riva E.G., Olmo M., Poorter H., Ubera J.L., Villar R. (2016). Leaf mass per area (LMA) and its relationship with leaf structure and anatomy in 34 Mediterranean woody species along a water availability gradient. PLoS ONE.

[50] Farquharson K.L. (2017). Fine-tuning plant growth in the face of drought. Plant Cell.

[51] Koch G., Rolland G., Dauzat M., Bédiée A., Baldazzi V., Bertin N., Guédon Y., Granier C. (2019). Leaf production and expansion: A generalized response to drought stresses from cells to whole leaf biomass—A case study in the tomato compound leaf. Plants.

[52] Lehmeier C., Pajor R., Lundgren M.R., Mathers A., Sloan J., Bauch M., Mitchell A., Bellasio C., Green A., Bouyer D. (2017). Cell density and airspace patterning in the leaf can be manipulated to increase leaf photosynthetic capacity. Plant. J..

[53] Lee L.R., Bergmann D.C. (2019). The plant stomatal lineage at a glance. J. Cell Sci..

[54] Rozema J., Björn L.O., Bornman J.F., Gaberščik A., Häder D.P., Trošt T., Germ M., Klisch M., Gröniger A., Sinha R.P. (2002). The role of UV-B radiation in aquatic and terrestrial ecosystems-an experimental and functional analysis of the evolution of UV-absorbing compounds. J. Photochem. Photobiol. B Biol..

[55] Falcone Ferreyra M.L., Rius S.P., Casati P. (2012). Front. Flavonoids: Biosynthesis, biological functions, and biotechnological applications. Front. Plant Sci..

[56] Mathesius U. (2018). Flavonoid functions in plants and their interactions with other organisms. Plants.

[57] Basahi M.J., Ismail I.M., Hassan I.A. (2014). Effects of enhanced UV-B radiation and drought stress on photosynthetic performance of lettuce (*Lactuca sativa* L. Romaine) plants. Annu. Res. Rev. Biol..

[58] Nakabayashi R., Mori T., Saito K. (2014). Alternation of flavonoid accumulation under drought stress in Arabidopsis thaliana. Plant Signal. Behav..

[59] Hussain M., Farooq S., Hasan W., Ul-Allah S., Tanveer M., Farooqh M., Nawaz A. (2018). Drought stress in sunflower: Physiological effects and its management through breeding and agronomic alternatives. Agric. Water Manag..

[60] Marschner H. (1995). Mineral Nutrition of Higher Plants.

[61] Franceschi V.R., Nakata P.A. (2005). Calcium oxalate in plants: Formation and function. Annu. Rev. Plant Biol..

[62] Ustin S.L., Jacquemoud S., Govaerts Y. (2001). Simulation of photon transport in a three-dimensional leaf: Implications for photosynthesis. Plant Cell Environ..

[63] Klančnik K., Mlinar M., Gaberščik A. (2012). Heterophylly results in a variety of “spectral signatures” in aquatic plant species. Aquat. Bot..

[64] Brotherton S.J., Joyce C.B. (2015). Extreme climate events and wet grasslands: Plant traits for ecological resilience. Hydrobiologia.

[65] Gutschick V.P., BassiriRad H. (2003). Extreme events as shaping physiology, ecology, and evolution of plants: Toward a unified definition and evaluation of their consequences. New Phytol..

[66] Volk G.M., Lynch-Holm V.J., Kostman T.A., Goss L.J., Franceschi V.R. (2002). The role of druse and raphide calcium oxalate crystals in tissue calcium regulation in *Pistia stratiotes* leaves. Plant Biol..

[67] Osakabe Y., Osakabe K., Shinozaki K., Tran L.-S.P. (2014). Response of plants to water stress. Front. Plant Sci..

