# Supplementary material for: Water Shortage Strongly Alters Formation of Calcium Oxalate Druse Crystals and Leaf Traits in Fagopyrum esculentum
Source: Plants (Basel). 2020 Jul 20;9(7):917. doi: 10.3390/plants9070917 (PMC7411882; doi:10.3390/plants9070917)
Supplement: Supplementary file 1 [file plants-09-00917-s001.zip › plants-855643-supplementary/Supplement_1.docx]

**Supplementary Materials:**

**Table S1.** Meteorological conditions during the field experiment.

| **LJUBLJANA - BEŽIGRAD lon=14.5124 lat=46.0655 altitude=299 m** | **Mean Day T [°C]** | **Mean Wind [m/s]** | **Mean Relative Humidity [%]** | **Rainfall [mm]** | **Solar Iradiation [h]** |
| --- | --- | --- | --- | --- | --- |
| **2018-05-23** | 19.4 | 1 | 82 | 6.9 | 3 |
| **2018-05-24** | 19.7 | 0.6 | 80 | 0 | 3.8 |
| **2018-05-25** | 20 | 1.3 | 74 | 0 | 8 |
| **2018-05-26** | 21.6 | 1.1 | 72 | 0 | 9.8 |
| **2018-05-27** | 20 | 1.2 | 74 | 0 | 8.9 |
| **2018-05-28** | 23 | 0.6 | 67 | 0.3 | 9.6 |
| **2018-05-29** | 19.7 | 0.7 | 83 | 0 | 4.1 |
| **2018-05-30** | 21.6 | 0.9 | 78 | 8.5 | 5.7 |
| **2018-05-31** | 23.4 | 1.5 | 65 | 0 | 6.2 |
| **2018-06-01** | 22.9 | 1.2 | 62 | 0 | 11.6 |
| **2018-06-02** | 20.6 | 1 | 66 | 0 | 10.6 |
| **2018-06-03** | 23.1 | 0.8 | 60 | 4 | 13.6 |
| **2018-06-04** | 23.6 | 1.3 | 57 | 0 | 13 |
| **2018-06-05** | 17.6 | 1.6 | 87 | 0 | 6.6 |
| **2018-06-06** | 21.6 | 2.1 | 69 | 13.8 | 9 |
| **2018-06-07** | 21.3 | 0.9 | 74 | 0 | 4.8 |
| **2018-06-08** | 20.4 | 1.5 | 78 | 0 | 3.4 |
| **2018-06-09** | 21.8 | 1.4 | 75 | 9.9 | 9.1 |
| **2018-06-10** | 24.2 | 0.8 | 66 | 0 | 10 |
| **2018-06-11** | 25.2 | 1.8 | 64 | 0 | 9.8 |
| **2018-06-12** | 22.8 | 1.4 | 68 | 0 | 7.4 |
| **2018-06-13** | 19.1 | 1.5 | 77 | 1.7 | 5.9 |
| **2018-06-14** | 18.8 | 1.5 | 64 | 0.3 | 3.7 |
| **2018-06-15** | 20.1 | 1.6 | 56 | 0 | 6.6 |
| **2018-06-16** | 21 | 1 | 57 | 0 | 11.2 |
| **2018-06-17** | 20.3 | 0.9 | 68 | 0 | 3.9 |
| **2018-06-18** | 23.1 | 0.7 | 57 | 0 | 11.2 |
| **2018-06-19** | 23.4 | 0.6 | 53 | 0 | 12.2 |
| **2018-06-20** | 24.4 | 0.5 | 58 | 0 | 13.6 |
| **2018-06-21** | 25.3 | 2.2 | 57 | 0 | 12.7 |
| **2018-06-22** | 14.8 | 3.2 | 84 | 22.7 | 1.8 |
| **2018-06-23** | 17.1 | 1 | 60 | 15.9 | 9.6 |
| **2018-06-24** | 17.7 | 0.7 | 69 | 0.1 | 7.2 |
| **2018-06-25** | 16.4 | 1.6 | 75 | 7 | 3.8 |
| **2018-06-26** | 17.8 | 0.8 | 67 | 4.4 | 7.9 |
| **2018-06-27** | 19.1 | 0.2 | 62 | 0.4 | 7.8 |
| **2018-06-28** | 19.1 | 1.5 | 77 | 2 | 1.1 |
| **2018-06-29** | 22.9 | 1.1 | 62 | 0.7 | 5.1 |
| **2018-06-30** | 22.7 | 2.1 | 70 | 0.6 | 8.5 |
| **2018-07-01** | 18.6 | 1 | 55 | 0.1 | 3.3 |
| **2018-07-02** | 20.8 | 0.4 | 67 | 0 | 1.8 |
| **2018-07-03** | 20.7 | 0.7 | 80 | 0 | 3.8 |
| **2018-07-04** | 22.3 | 0.2 | 77 | 30.8 | 5.8 |
| **2018-07-05** | 24.1 | 1 | 67 | 0 | 7.8 |
| **2018-07-06** | 21.1 | 0.5 | 75 | 1.4 | 2.3 |
| **2018-07-07** | 22.8 | 0.6 | 64 | 0 | 11.1 |
| **2018-07-08** | 20.2 | 0.9 | 68 | 0 | 9.6 |
| **2018-07-09** | 21.9 | 1.1 | 59 | 2 | 9.2 |

Source: <https://meteo.arso.gov.si/met/sl/archive/>
