# Supplementary material for: Water Shortage Strongly Alters Formation of Calcium Oxalate Druse Crystals and Leaf Traits in Fagopyrum esculentum
Source: Plants (Basel). 2020 Jul 20;9(7):917. doi: 10.3390/plants9070917 (PMC7411882; doi:10.3390/plants9070917)
Supplement: Supplementary file 1 [file plants-09-00917-s001.zip › plants-855643-supplementary/Supplement_2.docx]

**Supplementary Materials:**

**Figure S1.** RDA plot showing the strength of associations of thickness of palisade mesophyll and tissue density with druses of CaOx crystals parameters. Dark grey circles, control; white circles, water shortage.
