# Supplementary material for: Water Shortage Strongly Alters Formation of Calcium Oxalate Druse Crystals and Leaf Traits in Fagopyrum esculentum
Source: Plants (Basel). 2020 Jul 20;9(7):917. doi: 10.3390/plants9070917 (PMC7411882; doi:10.3390/plants9070917)
Supplement: Supplementary file 1 [file plants-09-00917-s001.zip › plants-855643-supplementary/Supplement_3.docx]

**Supplementary Materials:**

(A)

(B)

**Figure S2.** Reflectance (**A**) and transmittance (**B**) spectra of control and water-deprived buckwheat leaves. C, control; W-, water shortage.
